# Integrated transcriptomic analysis identifies coordinated responses to nitrogen and phosphate deficiency in rice

**DOI:** 10.3389/fpls.2023.1164441

**Published:** 2023-05-08

**Authors:** Fei Wang, Yan Wang, Luying Ying, Hong Lu, Yijian Liu, Yu Liu, Jiming Xu, Yunrong Wu, Xiaorong Mo, Zhongchang Wu, Chuanzao Mao

**Affiliations:** ^1^ State Key Laboratory of Plant Physiology and Biochemistry, College of Life Sciences, Zhejiang University, Hangzhou, China; ^2^ Hainan Institute, Zhejiang University, Yazhou Bay Science and Technology City, Sanya, Hainan, China

**Keywords:** rice, nitrogen, phosphate, interaction, uptake, transcriptome, NIGT1

## Abstract

Nitrogen (N) and phosphorus (P) are two primary components of fertilizers for crop production. Coordinated acquisition and utilization of N and P are crucial for plants to achieve nutrient balance and optimal growth in a changing rhizospheric nutrient environment. However, little is known about how N and P signaling pathways are integrated. We performed transcriptomic analyses and physiological experiments to explore gene expression profiles and physiological homeostasis in the response of rice (*Oryza sativa*) to N and P deficiency. We revealed that N and P shortage inhibit rice growth and uptake of other nutrients. Gene Ontology (GO) analysis of differentially expressed genes (DEGs) suggested that N and Pi deficiency stimulate specific different physiological reactions and also some same physiological processes in rice. We established the transcriptional regulatory network between N and P signaling pathways based on all DEGs. We determined that the transcript levels of 763 core genes changed under both N or P starvation conditions. Among these core genes, we focused on the transcription factor gene *NITRATE-INDUCIBLE, GARP-TYPE TRANSCRIPTIONAL REPRESSOR 1* (*NIGT1*) and show that its encoded protein is a positive regulator of P homeostasis and a negative regulator of N acquisition in rice. NIGT1 promoted Pi uptake but inhibited N absorption, induced the expression of Pi responsive genes *PT2* and *SPX1* and repressed the N responsive genes *NLP1* and *NRT2.1*. These results provide new clues about the mechanisms underlying the interaction between plant N and P starvation responses.

## 1 Introduction

Phosphorus (P) and nitrogen (N) are two of the most important essential mineral elements for plant growth and are also the most widely used components of fertilizers that cause environmental pollution. The uptake and utilization of N and P by plants are often mutually affected (Gusewell, 2004), with changes in the N:P supply ratio significantly affecting their absorption (Luo et al., 2016). Inorganic phosphate (Pi) and nitrate ( ${\text{NO}}_{3}^{-}$ ) and ammonium ( ${\text{NH}}_{4}^{+}$ ) are the main forms of P and N taken up by most plant species. Plants have evolved similar regulatory networks to adapt to soil environments with different P and N nutrient contents, known as Pi and N starvation responses, respectively. The underlying pathways involve inducing the expression of genes encoding transporters, recycling internal P and N pools, changing root system architecture, and interacting with soil microbes (Bouain et al., 2019, Li et al., 2021b). Previous studies have mainly focused on P and N signal transduction pathways separately (Wang et al., 2018, Li et al., 2021b, Lu et al., 2022; Paz-Ares et al., 2022), and very few have examined the mutual regulation of the uptake and utilization of these two nutrient elements.

Recent studies have revealed several genes that regulate P–N interactions in plants. Arabidopsis (*Arabidopsis thaliana*) *NITROGEN LIMITATION ADAPTATION* (*NLA*) encodes an E3 ligase that regulates the protein stability of the nitrate transporter NRT1/PTR FAMILY 2.13 (NPF2.13, also named NITRATE TRANSPORTER 1.7 [NRT1.7]) and PHOSPHATE TRANSPORTER 1 (PHT1), resulting in N-dependent P accumulation in shoots (Kant et al., 2011; Lin et al., 2013; Park et al., 2014; Liu et al., 2017). Rice (*Oryza sativa*) NLA1 mediates the degradation of the phosphate transporters PT2 and PT8 to regulate P acquisition (Yang et al., 2017; Yue et al., 2017). Rice NITRATE-INDUCIBLE, GARP-TYPE TRANSCRIPTIONAL REPRESSOR 1 (NIGT1) is a nitrate-induced GARP-type transcription factor (TF) that binds 5′-GAATC-3′ and 5′-GATATTC-3′ sequences to regulate the expression of downstream genes (Sawaki et al., 2013); however, its biological function has not been studied. Arabidopsis contains four *NIGT1* homologs that affect root growth and plant P and N uptake by regulating the expression of *SPX1* (*SYG1/Pho81/XPR1*), *PHT*, and *NRT* genes (Medici et al., 2015; Kiba et al., 2018; Maeda et al., 2018, Ueda et al., 2020a; Wang et al., 2020b). In addition, studies have shown that rice NRT1.1B can interact with SPX4 to promote its degradation under high nitrate conditions, thereby relieving the inhibition by SPX4 of nuclear translocation for the Pi signaling regulator PHOSPHATE STARVATION RESPONSE 2 (PHR2) and the N signaling regulator NIN-LIKE PROTEIN 3 (NLP3) (Hu et al., 2019). Studies in Arabidopsis have shown that Pi deficiency promotes ammonium assimilation mediated by AMT1 transporters, accompanied by H^+^ expulsion, activates STOP1 (SENSITIVE TO PROTON RHIZOTOXICITY 1) and increases organic acid secretion, thereby releasing Pi from Fe (and Al) containing complexes (Tian et al., 2021). At the same time, STOP1 can directly bind to the promoter of *NRT1.1* to activate its transcription at low pH, therefore promote ${\text{NO}}_{3}^{-}$ uptake (Ye et al., 2021).

In response to low external P and N, plants undergo global physiological changes and transcriptome reprogramming (Ueda et al., 2020c). Transcriptome analysis is a rapid and effective method for studying regulatory networks, allowing us to understand plant stress responses comprehensively and identify potential functional genes, including TF genes and their targets (Ueda et al., 2020c, Nie et al., 2021; Yan et al., 2021; Li et al., 2022). For example, time-series transcriptome analyses revealed CYTOKININ RESPONSE FACTOR 4 (CRF4) as an early player in mediating the N response in Arabidopsis (Varala et al., 2018). Comparative omics analysis also provides a new approach for completely understanding the mechanisms underlying specific physiological responses (Liang et al., 2022; Li et al., 2022; Zhou et al., 2022). Although several genes associated with N and P acquisition have been identified in rice, the precise molecular mechanism behind N–P interaction remains elusive.

In this study, we conducted combined transcriptomic analyses of rice roots responding to N or Pi deficiency to identify genes associated with both N and P homeostasis in rice. Our results highlighted many genes shared by the N and P starvation responses. We identified a list of candidate core genes that may coordinate N and P responses and metabolisms. Furthermore, we functionally identified rice NIGT1 as one such integrator of N and P homeostasis.

## 2 Materials and methods

### 2.1 Plant materials and growth conditions

Two wild-type *japonica* rice (*Oryza sativa* L.) cultivars, ‘Nipponbare’ and ‘Hei Jing 2’ (HJ2), were used in this study. Nipponbare plants were used for transcriptome analysis. All transgenic rice lines were produced in the HJ2 background. Rice plants were grown hydroponically in a growth chamber at 30/22°C (day/night), 60 to 70% humidity, with a light intensity of ~300 μmol m^–2^ s^–1^ provided by a bulb-type light and a photoperiod of 14 h light/10 h dark, as previously described (Wang et al., 2020a). The nutrient solution was adjusted to pH 5.5 using 1 M HCl or NaOH. Concentrations of KH_2_PO_4_ and NH_4_NO_3_ were adjusted to 200/1,400, 10/1,400, 0/1,400, 200/70, and 200 μM/0 μM for control (CK), low-P (LP), P deficiency (–P), low-N (LN), and N deficiency (–N) experimental conditions, respectively. The other macronutrients are K_2_SO_4_ (191.5 μM), CaCl_2_·2H_2_O (366 μM) and MgSO_4_·7H_2_O (547 μM). The nutrient solution also contained the following micronutrients: MnCl_2_·4H_2_O (0.0005 μM), H_3_BO_3_ (0.003 μM), (NH_4_)_6_Mo_7_O_24_·4H_2_O (0.0001 μM), ZnSO4·7H2O (0.0004 μM), CuSO4·5H2O (0.0002μM) and NaFe(3)-EDTA (40 μM).

### 2.2 Nutrient content measurements

All plants (the wild type and T2 transgenic lines) were harvested after treatment, separated into roots and shoots, and then dried at 70°C for 3 d. Tissues were weighed and digested with 98% H_2_SO_4_ (v/v) and 30% H_2_O_2_ (v/v) at 300°C. Total N content was determined using the Kjeldahl N assay (Xia et al., 2015). Both N and P determination were conducted using a continuous flow analyzer (SKALAR, SKALAR San plus system) as previously described (Wang et al., 2020a). Potassium (K), calcium (Ca), and magnesium (Mg) concentrations were determined by inductively coupled plasma–optical emission spectrometry (Yang et al., 2016).

### 2.3 RNA-seq and gene expression analysis

Fourteen-day-old Nipponbare seedlings cultured in full-strength nutrient solution were transferred to –N and –P solutions and cultured for a further 7 d. Roots from three to four independent plants were harvested, pooled together as one biological replicate, and used for RNA-seq. Three biological repeats were performed per treatment. RNA-seq experiments were performed by Novogene Bioinformatics Technology Co. Ltd (China). Total RNA was extracted using TRIzol reagent (Invitrogen). mRNA libraries were constructed according to the Illumina instructions and sequenced using a HiSeq 2500 instrument; all paired-end reads were mapped to the rice cv Nipponbare genome (http://ftp.ensemblgenomes.org/pub/release-24/plants/fasta/oryza_sativa/dna/Oryza_sativa.IRGSP-1.0.24.dna.toplevel.fa.gz) using TopHat (v2.0.12). All raw sequence read data were uploaded to the NCBI Sequence Read Archive under accession number PRJNA925220.

The DESeq R package (v1.10.1) was used for analyzing differentially expressed genes (DEGs). Thresholds were set as a false discovery rate (FDR) adjusted *P* < 0.05 (*P* values were adjusted using the Benjamini and Hochberg method) and an absolute value of Log_2_(fold-change) > 1 to determine significant differences in gene expression. Clustered genes were assigned to biological process categories based on Gene Ontology (GO) analysis using the web tool THE GENE ONTOLOGY RESOURCE (http://geneontology.org/). GO terms with FDR < 0.05 were considered significantly enriched for DEGs. Heatmaps of DEGs were visualized using TBtools (Chen et al., 2020). TF IDs were downloaded from the database PlantTFDB (http://planttfdb.gao-lab.org/). Predicted regulatory networks between enriched TFs and their potential target genes were identified using the PlantRegMap database (http://plantregmap.gao-lab.org/tf_enrichment.php).

### 2.4 RT‐qPCR analysis

Total RNA was isolated from rice samples using TRIzol reagent (Invitrogen). First-strand cDNA was synthesized from 1 µg of total RNA using a reverse transcription system (A3500; Promega). qPCR was performed using SYBR Green I Master (Roche) on a LightCycler 480 Real-Time PCR system (Roche) according to the manufacturer’s instructions. Relative expression levels were normalized to those of the housekeeping gene *ACTIN1* (Os03g0718100), as previously described (Wang et al., 2020a). Primers used for qPCR are listed in **Table S1**.

### 2.5 Vector construction and generation of transgenic plants

To generate *nigt1* knockout mutants, two specific targets at different locations in the *NIGT1* genomic sequence were selected for creating single guide RNAs (sgRNAs). The target sequences were ligated into the pYLCRISPR/gRNA vector, followed by ligation into the pYLCRISPR/Cas9-MH vector as described (Shao et al., 2019). To generate *35S::NIGT1-GFP* overexpression lines, the full-length *NIGT1* (1236 bp) coding sequence was amplified by PCR from cDNA using primer pair NIGT-GFP-F and NIGT-GFP-R and introduced into a modified pCAMBIA1300-35S-eGFP plasmid using *Bam*HI and *Xba*I restriction sites downstream of the 35S promoter. All constructs were confirmed by sequencing. All transgenic plants were produced *via* Agrobacterium (*Agrobacterium tumefaciens*) (strain EHA105)-mediated transformation. Primers used for vector construction are listed in **Table S1**.

### 2.6 Subcellular localization

*35S::NIGT1-GFP* was transiently expressed into *Nicotiana benthamiana* leaves by Agrobacterium-mediated infiltration. Fluorescence in roots of 7-d-old transgenic rice seedlings harboring the *35S::NIGT1-GFP* transgene was observed using a confocal laser-scanning microscope (Axiovert LSM 710; Zeiss). A×25 water immersion objective was used for confocal imaging. Fluorescent proteins were excited at 488 nm using an argon ion laser. Fluorescence was detected from 493 to 542 nm.

### 2.7 Statistical analyses

Statistical analysis was conducted using the program SPSS Statistics v22.0 (IBM).

## 3 Results

### 3.1 Nitrogen or phosphate deficiency affects rice growth and N/P homeostasis

**Figure 1:**
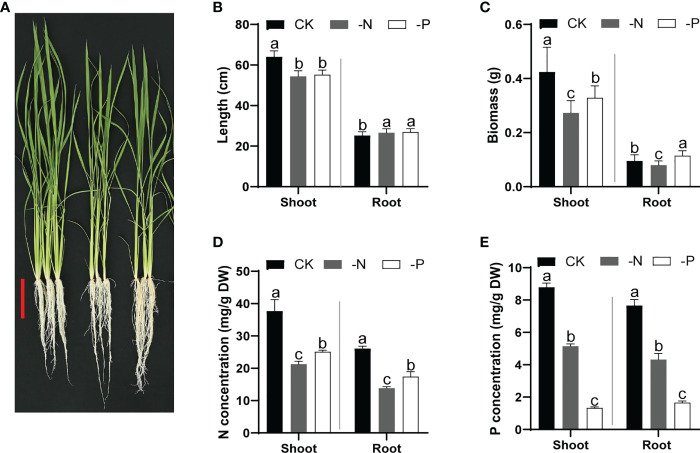
Growth performance and N/P content of rice plants cultured under different nutrient conditions. **(A)** Growth phenotypes of wild-type Nipponbare in different nutrient solutions. Fourteen-day-old seedlings cultured in replete nutrient solution were transferred to –N or –P solutions and cultured for another 14 d. Scale bar, 10 cm. **(B–E)** Shoot and root length **(B)**, biomass **(C)**, N concentration **(D)**, and P concentration **(E)** of rice seedlings shown in **(A)** Data are means + SD (*n* = 30 in **(B, C)**, *n* = 4 in **(D, E)**). Different lowercase letters indicate significant differences between groups (ANOVA followed by Tukey’s test, *P* < 0.05).

To confirm the effects of N and Pi deficiency on rice development, we cultured wild-type Nipponbare seedlings in normal nutrient solution (CK) for 14 d and then transferred them to CK, –P (no Pi), or –N (no N) solutions for another 14 d. Both –P and –N conditions inhibited rice shoot growth but promoted root elongation (**Figures 1A, B**) and resulted in a lower overall biomass. Furthermore, N deficiency led to a relatively more severe growth inhibition than P deficiency (**Figure 1C**). The SPAD value (for evaluating chlorophyll content) of rice leaves treated with –N was lower than that for the CK treatment, consistent with the yellowish leaves of N-deficient rice seedlings (**Figure 1A** and **Figure S1A**). Moreover, the N content of rice was lowest under N deficiency, and the P content was lowest under Pi deficiency, validating the deficiency conditions (**Figures 1D, E**). Furthermore, the P and N concentrations in shoots or roots of rice plants grown under either –N or –P conditions were lower than those of rice plants grown under normal conditions (**Figures 1D, E**). Plants grown under N deficiency showed lower K concentrations than those under CK conditions; plants grown under Pi deficiency displayed lower concentrations of K, Ca, and Mg (**Figures S1B-D**). These results indicate the mutual influence of N uptake and P acquisition, which is critical for optimizing plant growth under diverse nutrient conditions.

### 3.2 Profiling the transcriptional response to nitrogen and phosphate deficiency

**Figure 2:**
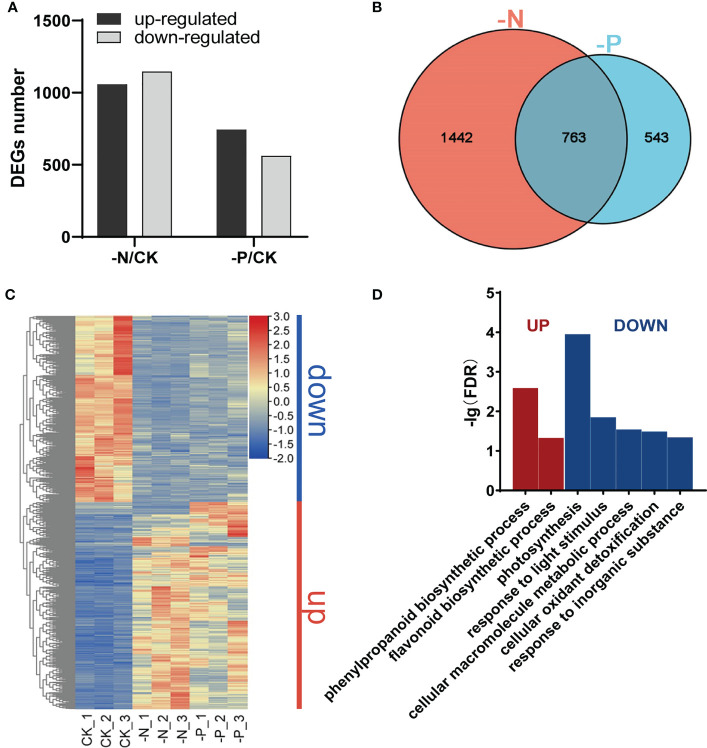
Changes in the rice transcriptome during N/P deficiency. **(A)** Numbers of upregulated and downregulated genes in Nipponbare exposed to –N or –P compared to normal conditions (CK). **(B)** Venn diagram of genes responsive to different N/P conditions. **(C)** Heatmap of DEGs responsive to both N and P deficiency; “down”, genes downregulated under –N or –P; “up”, genes upregulated under –N or –P. **(D)** Gene Ontology (GO) enrichment analysis of DEGs in **(C)**. Significant categories (FDR < 0.05) are displayed.

To obtain molecular insight into the transcriptional changes in response to N and P availability, we performed transcriptome deep sequencing (RNA-seq) on rice root tissues harvested after 7 d of treatment with N or Pi deficiency. The number of differentially expressed genes (DEGs) between the –N treatment and the control (CK) was much higher than that between the –P treatment and the CK treatment (2,205 vs. 1,306), with an adjusted *P* value (*p*-adj) < 0.05 and |Log_2_(fold-change)| ≥ 1 as cutoffs (**Figure 2A**). More DEGs were downregulated than upregulated in –N conditions relative to CK (1,147 vs. 1,058; **Figure 2A**; **Dataset S1**), while we detected more upregulated DEGs than downregulated DEGs in –P conditions compared to CK (744 vs. 562; **Figure 2A**; **Dataset S2**). DEGs triggered by –N or –P included known P or N starvation responsive genes, such as *SPX1* (Os06g0603600) (Wang et al., 2009), *PT2* (Os03g0150800) (Ai et al., 2009), *PT8* (Os10g0444700) (Jia et al., 2011), *NRT2.1* (Os02g0112100) (Takayanagi et al., 2011), *DEGRADATION OF UREA 3* (*DUR3*) (Os10g0580400) (Wang et al., 2012), and *NLP1* (Os03g0131100) (Alfatih et al., 2020), suggesting that the P and N deficiency treatments were successful. A Venn diagram between the DEGs obtained from individual deficiency conditions revealed 763 common DEGs between the –N treatment and the –P treatment, indicating that these genes are responsive to both N and P deficiency (hereafter referred to as NP-responsive DEGs; **Figure 2B**; **Dataset S3**). There were 1,442 DEGs only responding to N deficiency and 543 DEGs only responding to P deficiency (hereafter referred to as N-responsive DEGs and P-responsive DEGs, respectively; **Figure 2B**; **Dataset S3**).

We conducted a Gene Ontology (GO) enrichment analysis on these DEGs to determine their biological function (FDR-corrected *P* value < 0.05). We classified upregulated and downregulated DEGs under N deficiency into more than 20 functional categories in the biological process category (**Figure S2A**). DEGs upregulated under –N were mainly associated with nitrogen transport and metabolism, biosynthesis of secondary metabolites, and cell wall-related processes, such as ammonium transport, nitrate assimilation, plant-type secondary cell wall biogenesis, cutin biosynthetic process, and isoprenoid biosynthetic process (**Figure S2A**). DEGs downregulated under –N were enriched in photosynthesis-related terms, including photosynthesis, photosystem II stabilization, response to high-light intensity, and photosystem II repair, as well as terms related to glycometabolism and detoxification, including disaccharide biosynthetic process, fructose 1,6-bisphosphate metabolic process, and cellular oxidant detoxification (**Figure S2A**). Multiple photosynthesis-related terms showed significant enrichment, indicating that genes involved in photosynthesis are downregulated under N starvation, consistent with the yellowish leaf phenotype under N deficiency. DEGs upregulated under –P were enriched in cellular responses to phosphate starvation, phosphate ion transport, glycerol metabolic process, glycerophospholipid catabolic process, sulfolipid and glycolipid biosynthetic process, and cellular response to cold (**Figure S2B**). These results suggest that N and P deficiency induce different physiological reactions in plants.

A heatmap of the 763 NP-responsive DEGs showed that most of these genes respond similarly to N and P starvation. Genes upregulated under N deficiency were also upregulated under P deficiency, while genes downregulated under N deficiency were also downregulated under P deficiency (**Figure 2C**). These genes could be separated into two groups based on their expression patterns, “Up” and “Down.” Genes in the “Up” group were enriched in the phenylpropanoid and flavonoid biosynthetic pathways. Genes in the “Down” group were mainly enriched in photosynthesis, response to light stimulus, cellular macromolecule metabolic process, cellular oxidant detoxification, and response to inorganic substance categories (**Figure 2D**). These results demonstrate that some of the same physiological and biochemical reactions are stimulated in plants experiencing N or P deficiency.

### 3.3 Transcriptional regulatory network of rice nitrogen and phosphate deficiency responses

TFs are systematic regulators of global gene expression upon N and P deficiency. From our RNA-seq data, we identified 81 TF genes specifically responding to N, 19 TF genes specifically responding to P, and 34 TF genes responding to NP (**Figure S3A** and **Dataset S4**). These TF genes belonged to 28 families, with the N-responsive TF genes mainly in the basic helix-loop-helix (bHLH), MYB, NAC, and WRKY families, P-responsive TF genes mainly belonging to the ETHYLENE-RESPONSE FACTOR (ERF) family, and NP-responsive TF genes mainly comprising MYB and NAC family members (**Figure S3A**). To explore the potential transcriptional regulatory hierarchy among these DEGs, we constructed a transcriptional regulatory network of these TFs and DEGs considered as targets using PlantRegMap (Tian et al., 2020). This analysis produced a sub-network predominantly centered around 27 TFs and consisting of 2,005 regulatory relationships with 1,210 unique genes (**Figure S3B** and **Dataset S5**). These TFs and target genes were widely distributed among N-, NP-, and P-responsive DEGs. We noticed that several TFs target genes, including those encoding other TFs, from all three groups of DEGs. The TF targeting the most DEGs was ERF19, which is involved in responses to abscisic acid and salt stress (Huang et al., 2021). *ERF19* is also a P-responsive gene, suggesting a reciprocal influence of P and abscisic acid on plants. N-responsive TF MYB63, regulating the second most target genes (**Figure S3B**), is reported to regulate cellulose biosynthesis during secondary cell wall formation (Noda et al., 2015). This result is consistent with enrichment of the GO term cellulose biosynthesis under N deprivation (**Figure S2A**). Os02g0695200, encoding a MYB TF that has not yet been characterized, represented the NP-responsive TF targeting the most genes (**Figure S3B**).

**Figure 3:**
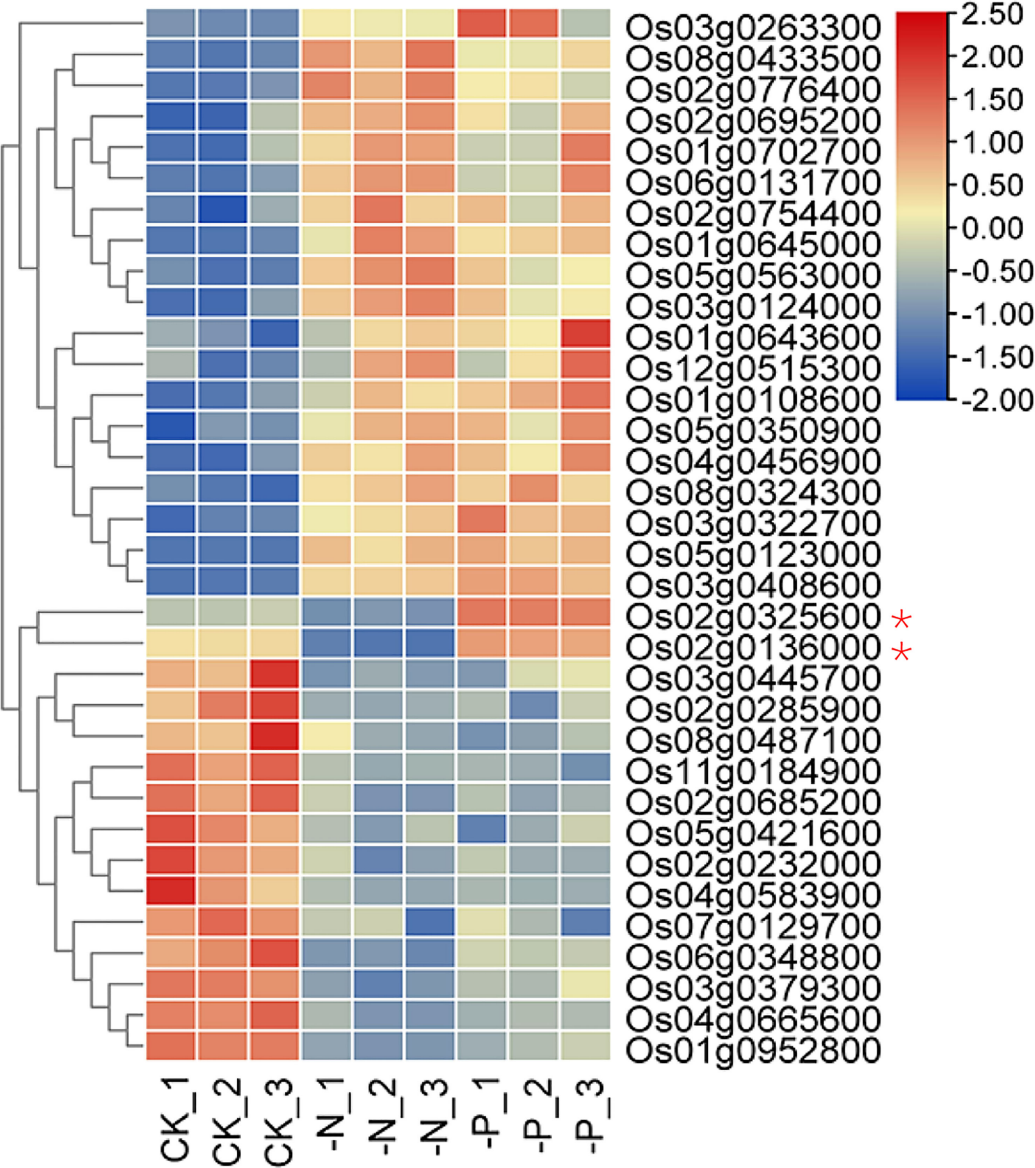
Heatmap of TF genes that respond to both N and P deficiency. Asterisks indicate genes with opposite responses to N and P deficiency.

The 34 NP-responsive TF genes belonged to 16 TF families, indicating that many types of TFs are involved in responses to both N and P deficiency (**Figure S3A**). MYB and MYB-related TFs formed the largest group. The central phosphate signaling regulator genes *AtPHR1* in Arabidopsis and *OsPHR2* in rice encode MYB family TFs that participate in nitrate-dependent phosphate signaling networks (Rubio et al., 2001; Zhou et al., 2008; Hu et al., 2019; Medici et al., 2019). Although the transcript levels of *AtPHR1* and *OsPHR2* are barely responsive to P deprivation, their homologs *AtPHL2*, *AtPHL3*, *OsPHR3*, and *OsPHR4* are upregulated upon P starvation (Sun et al., 2016; Ruan et al., 2017). The expression of most NP-responsive TFs (32 of 34) responded the same way to both N deficiency and P starvation (**Figures 2C** and **3**). Two genes showing opposite expression patterns under N and P deficiency were Os02g0325600 and Os02g0136000, encoding NIGT1 and NLP6, respectively. Whether these genes are involved in the response to N and P starvation awaits further study.

### 3.4 NIGT1 regulates nitrogen and phosphate homeostasis in rice

**Figure 4:**
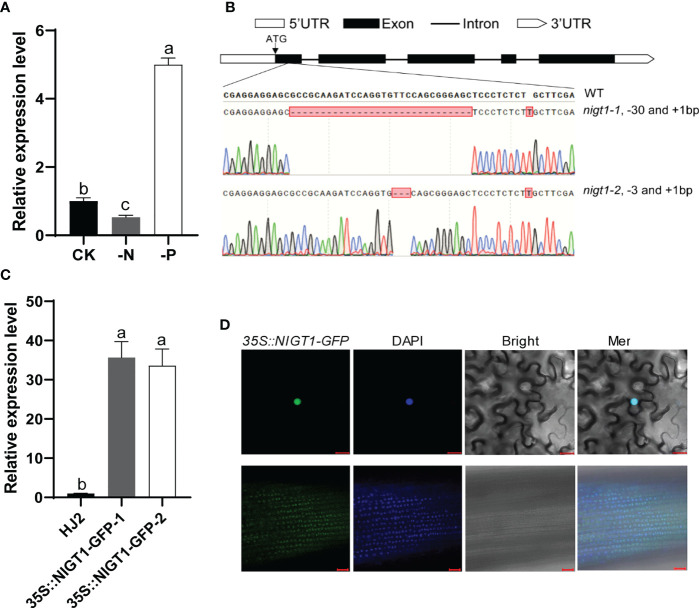
Molecular characterization of *NIGT1* mutants and transgenic overexpression lines. **(A)** Expression of *NIGT1* in HJ2 under –N or –P treatment for 7 d. Seedlings were grown under nutrient-replete hydroponic conditions for 7 d before –N or –P treatment. **(B)** Target sites and mutated sequences in *nigt1*-1 and *nigt1*-2 mutants generated using CRISPR/Cas9. **(C)** Expression of *NIGT1* in two *NIGT1*-overexpression transgenic lines. Seedlings were grown under nutrient-replete conditions for 2 weeks. *ACTIN1* was used as a control. *NIGT1* expression of the wild type under nutrient-replete conditions was set to 1. **(D)** GFP fluorescence in *N. benthamiana* leaf cells (upper panel) and root epidermal cells of 7-d-old transgenic rice seedlings (lower panel) harboring *35S::NIGT1-GFP*. Scale bars, 20 µm. Data are means + SD in **(A, C)** (*n* = 3); different lowercase letters indicate significant differences between groups (ANOVA followed by Tukey’s test, *P* < 0.05).

To verify whether *NIGT1* responds to N and P deficiency, we performed RT-qPCR analysis using total RNA isolated from the roots of rice plants exposed to N or P deficiency. Consistent with the results of RNA-seq analysis, *NIGT1* expression was induced by P deficiency and repressed by N deprivation (**Figure 4A**). To investigate the potential function of *NIGT1*, we generated multiple *nigt1* mutants using clustered regularly interspaced short palindromic repeat (CRISPR)/CRISPR-associated nuclease 9 (Cas9)-mediated genome editing to knock out *NIGT1* in the *japonica* rice cv. Hei Jing 2 (HJ2) and verified these by sequencing. We selected two independent lines carrying a 30- or 3-bp deletion and a 1-bp insertion in the first exon of the *NIGT1* gene, named *nigt1*-1 and *nigt1*-2, respectively, for further study (**Figure 4B**). The introduced mutations in both *nigt1*-1 and *nigt1*-2 resulted in premature stop codons and a predicted truncated protein. We also produced transgenic plants overexpressing *NIGT1* by ectopically expressing a *NIGT1*-*GFP* (green fluorescent protein) construct under the control of the cauliflower mosaic virus (CaMV) 35S promoter in HJ2. We chose two independent lines with the highest expression of *NIGT1-GFP* for subsequent experiments (**Figure 4C**). We observed a predominantly nuclear localization when *NIGT1-GFP* was transiently expressed in *N. benthamiana* leaves. Furthermore, we detected green fluorescence in *NIGT1-GFP* transgenic lines in the nuclei of root cells (**Figure 4D**), suggesting that NIGT1 is a nucleus-localized protein, consistent with NIGT1 functioning as a TF.

**Figure 5:**
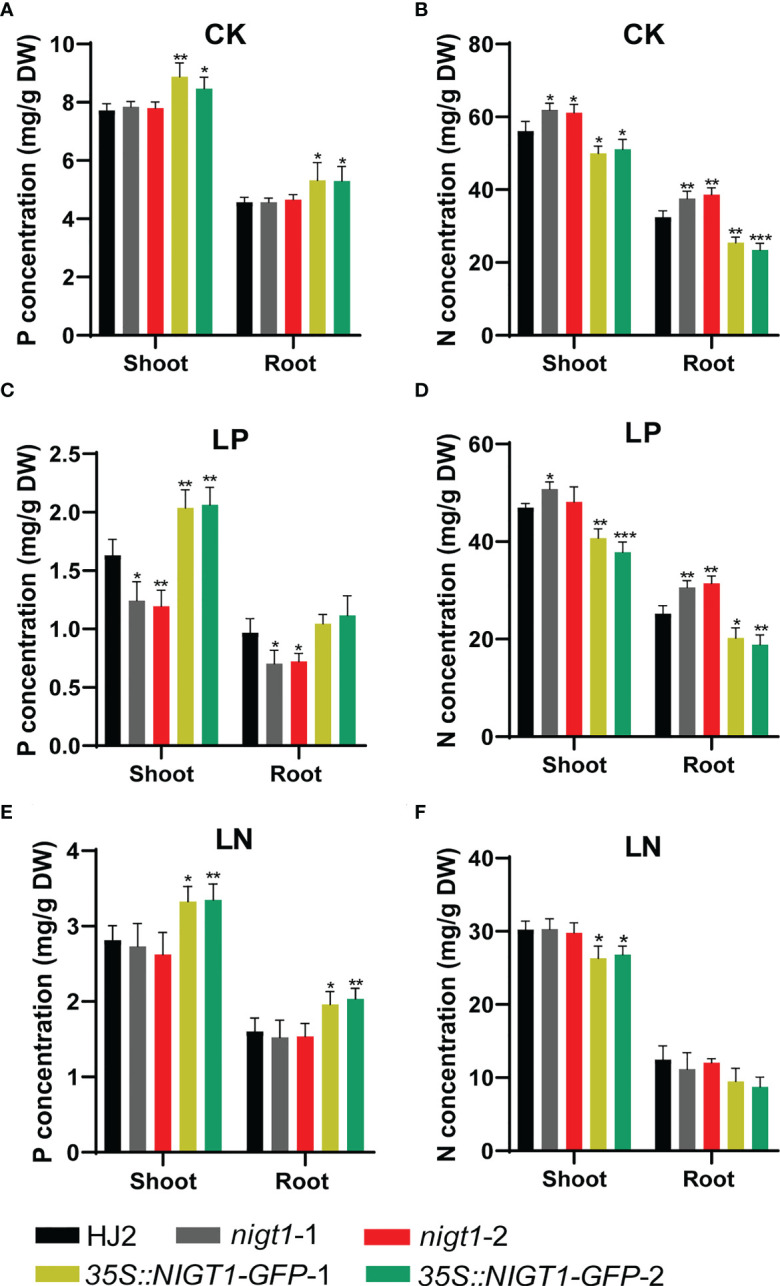
P and N concentrations in shoots and roots of rice *nigt1* mutants and *NIGT1* overexpression plants grown under different N and P conditions. **(A, C, E)** P concentration of plants. **(B, D, F)** N concentration of plants. Plants cultured in replete nutrient solution for 7 d were transferred to CK (normal N and P), LP (10 µM KH_2_PO_4_), or LN (70 µM NH_4_NO_3_) solutions and grown for another 21 d. Data are means + SD (*n* = 4). Student’s *t*-test *, *P* < 0.05; **, *P* < 0.01; ***, *P* < 0.001.

**Figure 6:**
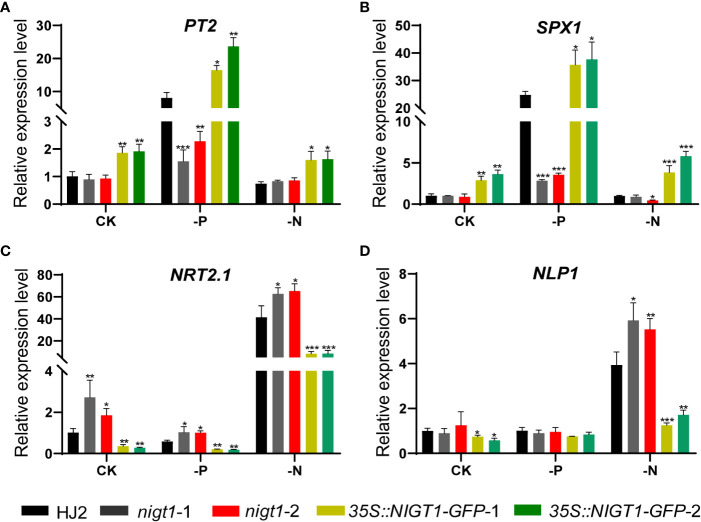
Expression levels of *PT2***(A)***, SPX1***(B)***, NRT2.1***(C)***, and NLP1***(D)** in HJ2, *nigt1* mutant, and *NIGT1* overexpression plants cultured under CK, LP, or LN conditions. *ACTIN1* was used as a control. Expression of the wild type HJ2 was set to 1. Data are means + SD (*n* = 3). Student’s *t*-test *, *P* < 0.05; **, *P* < 0.01; ***, *P* < 0.001.

To assess the involvement of NIGT1 in rice N and P homeostasis, we investigated N and P concentration of HJ2, the *nigt1* mutants, and *NIGT1* overexpression lines cultured in different nutrient solutions. Generally, accumulation of P in rice plants decreased under low-N supply; analogously, reducing the P supply also limited the levels of N in plant tissues (**Figures 1A** and **5**). Both *nigt1* mutants had lower P contents and higher N contents than wild-type plants, whereas *NIGT1* overexpression plants displayed higher P contents and lower N contents than wild-type plants (**Figure 5**), suggesting that NIGT1 promotes P uptake and inhibits N absorption. In addition, the shoots of *NIGT1* overexpression lines were shorter than those of wild-type plants, irrespective of growth conditions (**Figure S4**), indicating that *NIGT1* also affects rice shoot development. We asked whether NIGT1 mediated N and P signaling by measuring the expression levels of N- or P-responsive genes in the *nigt1* mutant and *NIGT1* overexpression lines. Transcript abundance of the N transporter gene *NRT2.1* was higher and that of the P transporter gene *PT2* was lower in *nigt1* mutant lines than in wild-type plants; by contrast, we detected lower *NRT2.1* and higher *PT2* transcript levels in *NIGT1* overexpression plants compared to wild-type plants (**Figures 6A, C**). In addition, *SPX1* accumulation induced by P deficiency was repressed and *NLP1* expression induced by N deficiency was enhanced in *nigt1* mutants compared to wild-type plants; we observed the opposite pattern in *NIGT1-*overexpressing lines (**Figures 6B, D**). These data demonstrate that N/P-responsive *NIGT1* plays a role in balancing the acquisition of nitrogen and phosphate in rice.

## 4 Discussion

Studying the mutual regulation of P and N signals is key for understanding the adaptive response of plants to a fluctuating soil environment; however, the mechanisms linking P and N acquisition in plant remain largely unknown. In this study, we investigated the similarities and differences of N and P deficiency on rice growth and transcript profiles. Both N and P deprivation inhibit shoot growth and mutually inhibit the uptake of P and N and even other nutrients in rice (**Figure 1** and **Figure S1**). All the mineral nutrient elements are absorbed *via* plants root. N or P deficiency changes the root system architecture (**Figures 1A, B**), which may affect the uptake of other elements. In consistency, the expression of transporter genes of K, Ca or Mg are changed upon N or P deficiency (**Datasets S1** and **S2**). However, deficiency in one of the microelements iron, zinc, or manganese significantly promotes the uptake of the other two elements (Dong et al., 2018), suggesting that the mutual regulatory mechanisms of different nutrient elements vary.

Rice is a typical species that prefers ammonium and lots of ammonium and nitrate coexist in the irrigated soil (Chen et al., 2018). It is of great importance to detect the combined effect of ammonium and nitrate in rice, so we used NH_4_NO_3_ as nitrogen source in this study. Nitrate deficiency can regulate the P starvation response in plants (Medici et al., 2019); however, whether P limitation affects the N starvation response remains elusive. Our comparative transcriptomic analysis revealed that one-third (763 out of 2,205) of the N-responsive DEGs accounted for more than one-half of all P-responsive DEGs (763 out of 1,306) (**Figure 2B**). These results indicate that low-N treatment can inhibit P accumulation and stimulate a P starvation response in plants. Similarly, P deficiency caused an N starvation response to some extent. The common N- and P-responsive genes were enriched in functions associated with phenylpropanoid and flavonoid biosynthesis, cellular oxidant detoxification, and response to inorganic substance, suggesting that plants will initiate basic defense mechanisms in response to N or P deficiency. Many genes were specifically responsive to either N deficiency or P deficiency. These results suggest that there are similarities and differences in plant responses to N and Pi deficiency.

Notably, many genes downregulated in roots were involved in photosynthesis, as evidenced by enriched GO categories such as response to light stimulus, photosystem II repair, photosynthesis, and light harvesting in photosystem I (**Figure 2D** and **Figure S2**). This observation suggests that genes involved in photosynthesis are also expressed in roots and that mobile signals contribute to the communication between roots and shoots. Photosynthesis involves many DNA, RNA and proteins that are rich in N or P (Hernandez and Munne-Bosch, 2015; Evans and Clarke, 2019; Mu and Chen, 2021), which may explain the downregulation of photosynthesis-related genes under N or P deficiency. Fluctuation in leaf N is strongly correlated with chlorophyll content (Hou et al., 2020); we observed lower chlorophyll content in rice plants exposed to N deficiency than in control plants, consistent with those genes associated with photosynthesis being affected by N deficiency. In addition, peptides, Ca^2+^, mRNAs, microRNAs, phytohormones, and nutrient ions themselves can act as mobile signals for long-distance communication between shoots and roots (Michigami et al., 2018; van Gelderen et al., 2018; Wang et al., 2018, Li et al., 2021a, Wheeldon and Bennett, 2021). Our RNA-seq data showed that the expression of the strigolactone (SL) biosynthesis genes *DWARF10* (*D10*) and *D27* was enhanced under –P and –N treatment (**Datasets S1** and **S2**). These results are consistent with previous results suggesting that low-phosphate and low-nitrate induce SL biosynthesis, mediating root growth (Mayzlish-Gati et al., 2012; Sun et al., 2014). Whether mobile signal molecules coordinate N and P homeostasis is worth further study.

Many TFs, especially PHR and NLP signaling regulators, regulate gene expression under N and P deficiency conditions (Bustos et al., 2010; Marchive et al., 2013; Guo et al., 2015; Gaudinier et al., 2018). We identified 27 TF-centered transcriptional regulatory networks from our RNA-seq data (**Figure S3**). Functional characterization of the TF gene *NIGT1* using gene-edited mutants and overexpression lines showed that *OsNIGT1*, which is induced by P starvation and repressed by N starvation (**Figure 4A**), negatively regulates N homeostasis and positively regulates P homeostasis (**Figure 5**). These results suggest that NIGT1 might play a role in the mutual inhibition of P uptake by N deficiency and of N uptake by P deprivation in rice. Expression levels of *PT2* and *SPX1* were lower in *nigt1* mutants and higher in *NIGT1* overexpression lines, while expression levels of *NRT2.1* and *NLP1* were higher in *nigt1* mutants and lower in *NIGT1* overexpression lines than those in wild-type plants (**Figure 6**). Furthermore, the promoters of these four genes contain a NIGT1-binding site (**Table S2**), indicating that they may be direct targets of NIGT1, which is worth confirming. In Arabidopsis, *NIGT1* (also named *HYPERSENSITIVITY TO LOW PI-ELICITED PRIMARY ROOT SHORTENING 1* [*HRS1*]) and *HRS1 HOMOLOG*s (*HHO*s) form a NIGT1-SPX–PHR signaling pathway and play an important role in coordinating N and P uptake and utilization during growth and development (Medici et al., 2015; Nagarajan et al., 2016; Kiba et al., 2018; Maeda et al., 2018, Ueda et al., 2020a, Ueda et al., 2020b). Recently, Wang et al. demonstrated that ZmNIGT1.1 and ZmNIGT1.2, two homologs of AtNIGT1.2, also participate in repression of ${\text{NO}}_{3}^{-}$ uptake under P deficiency in maize (*Zea mays*) (Wang et al., 2020b). These data indicate that the coordinated P–N regulatory pathway mediated by NIGT1 is conserved in monocot and dicot plants.

**Figure 7:**
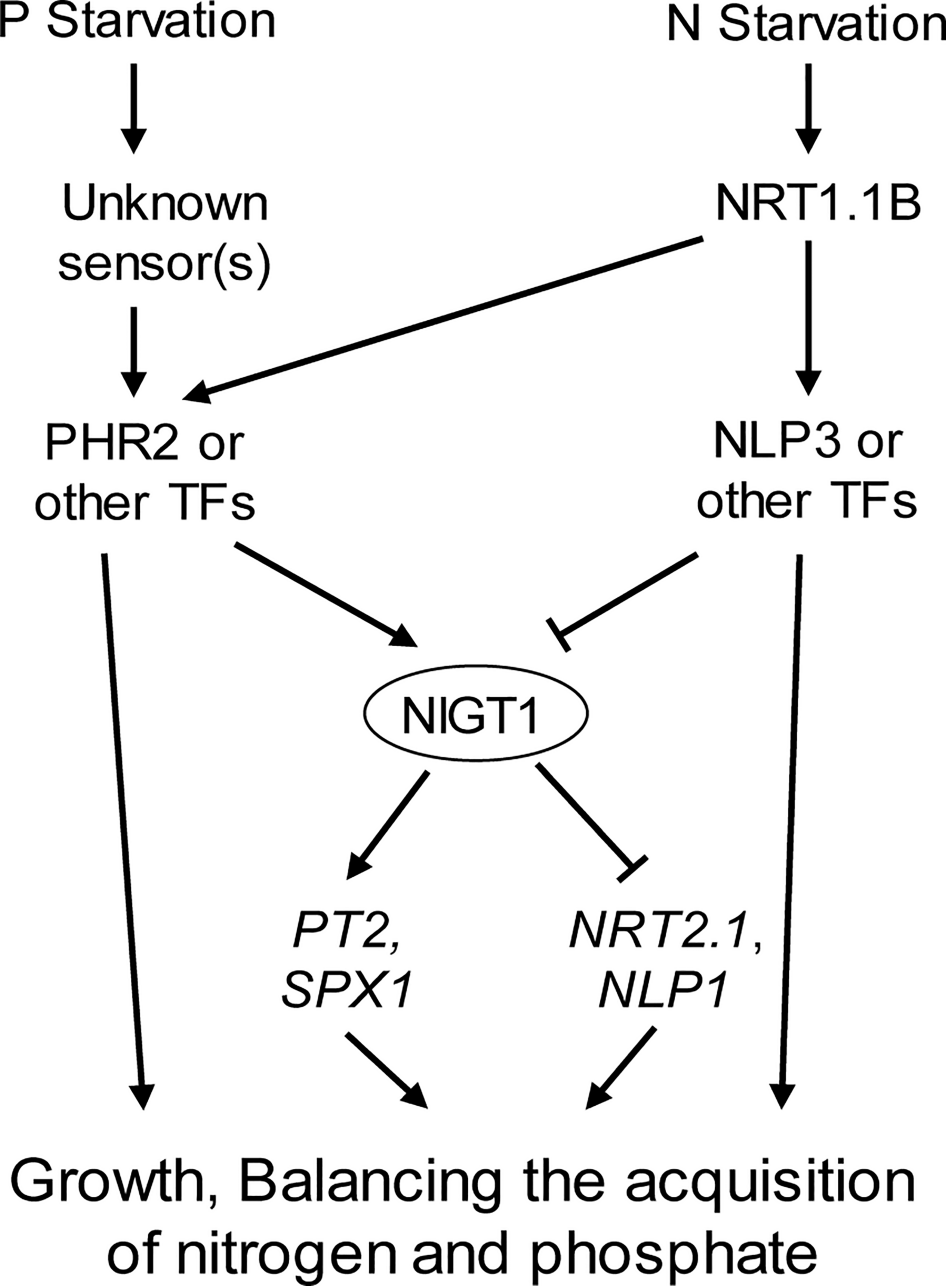
Proposed model for the regulation of plant responses to P and N deficiency. P and N starvation are perceived by respective sensors. The downstream signals are integrated by P deficiency induced and N starvation repressed TF NIGT1, which regulates *PT2*, *SPX1*, *NRT2.1*, and *NLP1* expression.

In summary, this work revealed the interaction between gene networks associated with N and P signaling in rice and the importance of NIGT1 in this interaction (**Figure 7**). These findings will help identify the N–P signaling interaction network and benefit breeding efforts to generate crops with higher N- and Pi-use efficiency.

## Data availability statement

The datasets presented in this study can be found in online repositories. The names of the repository/repositories and accession number(s) can be found below: https://www.ncbi.nlm.nih.gov/, PRJNA925220.

## Author contributions

CM and FW conceived and designed the experiments. FW performed the experiments. YW, LY, HL, YJL, YL, JX, and YRW helped with the experiments. XM and ZW revised the manuscript. CM and FW wrote the manuscript. All authors contributed to the article and approved the submitted version.
