# Supplementary material for: Integrated transcriptomic analysis identifies coordinated responses to nitrogen and phosphate deficiency in rice
Source: Front Plant Sci. 2023 May 8;14:1164441. doi: 10.3389/fpls.2023.1164441 (PMC10200874; doi:10.3389/fpls.2023.1164441)
Supplement: Supplementary file 1 [file Presentation_1.pptx]

## Slide 1
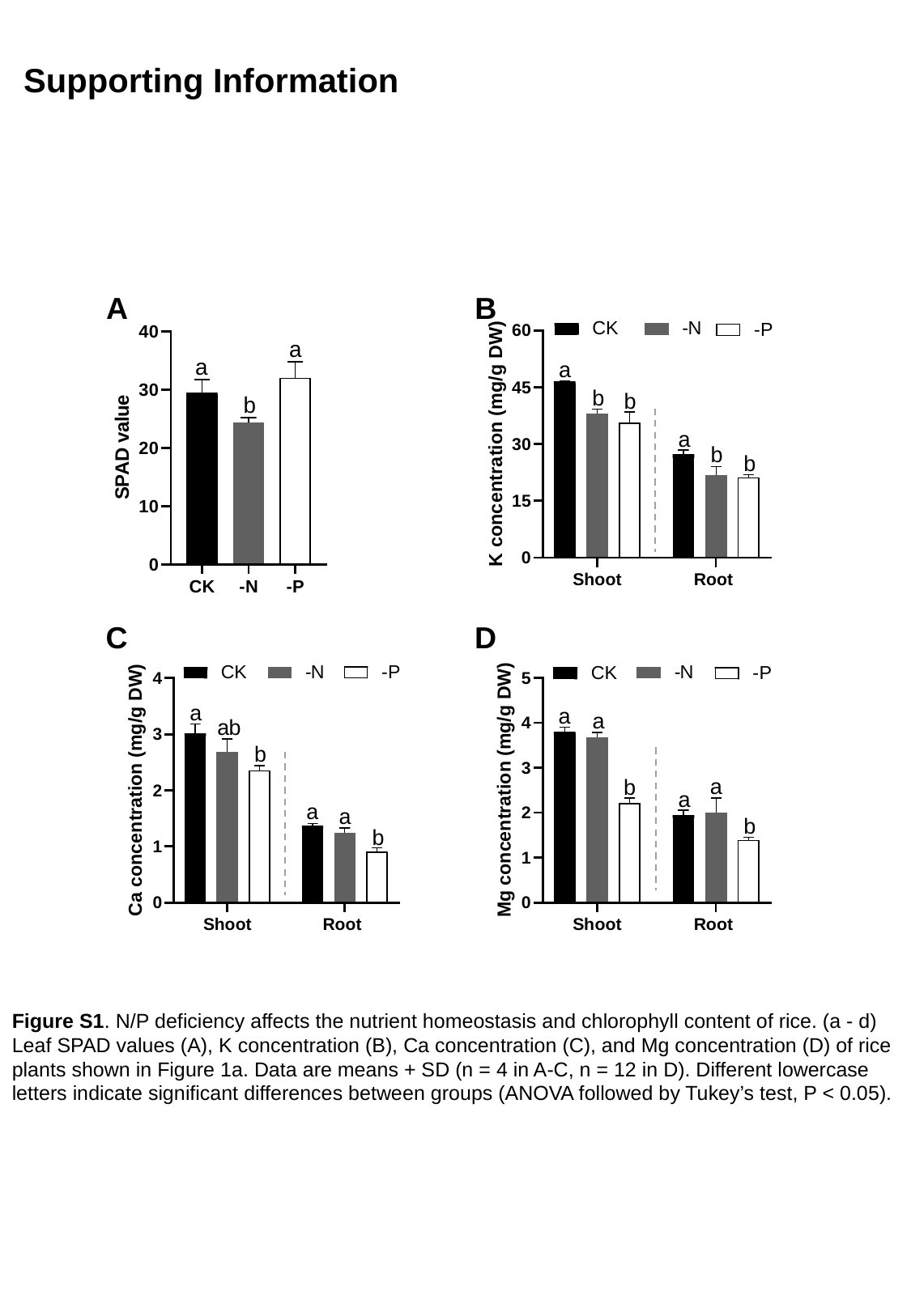

Supporting Information
A
B
C
D
Figure S1. N/P deficiency affects the nutrient homeostasis and chlorophyll content of rice. (a - d) Leaf SPAD values (A), K concentration (B), Ca concentration (C), and Mg concentration (D) of rice plants shown in Figure 1a. Data are means + SD (n = 4 in A-C, n = 12 in D). Different lowercase letters indicate significant differences between groups (ANOVA followed by Tukey’s test, P < 0.05).

## Slide 2
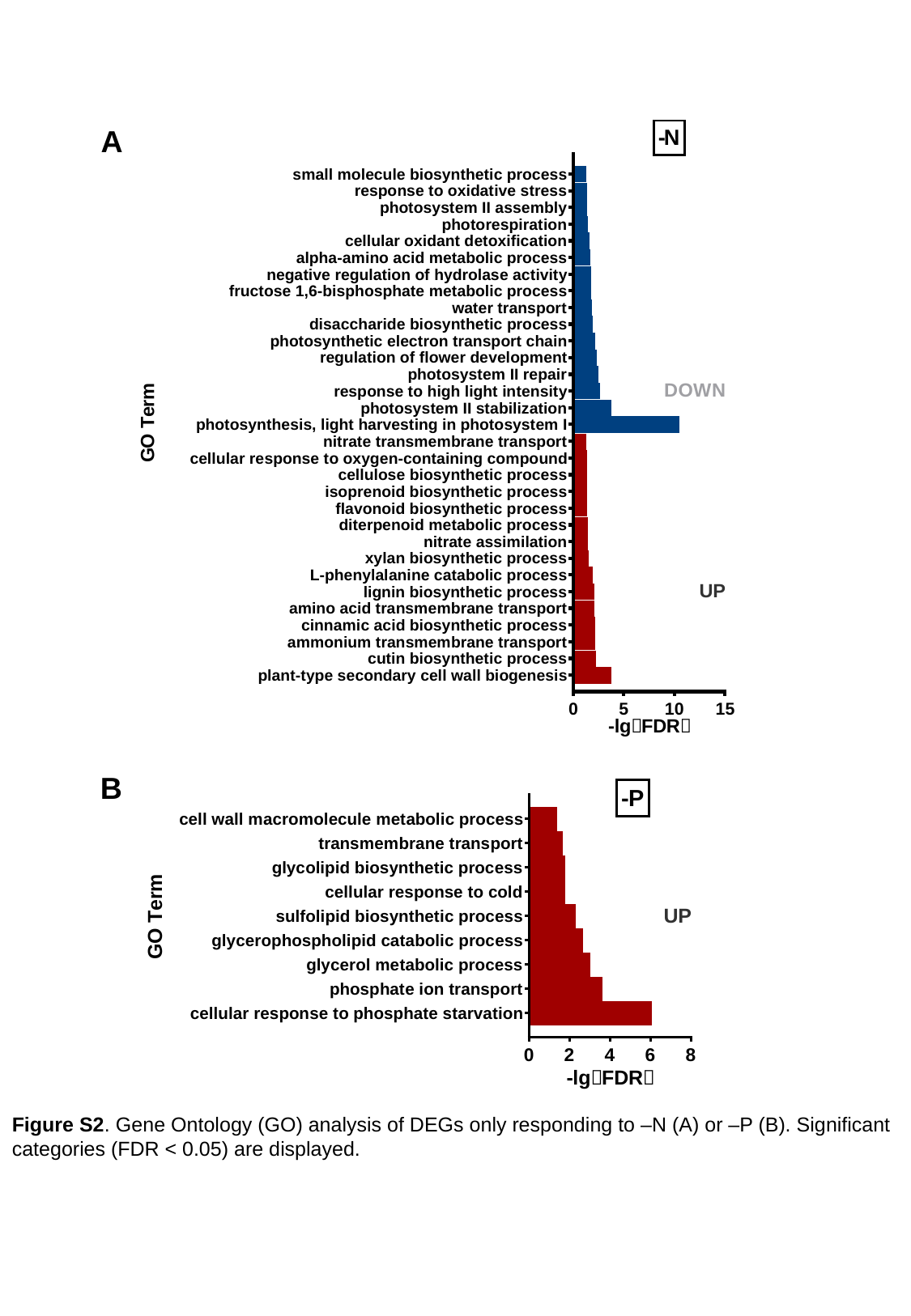

A
B
Figure S2. Gene Ontology (GO) analysis of DEGs only responding to –N (A) or –P (B). Significant categories (FDR < 0.05) are displayed.

## Slide 3
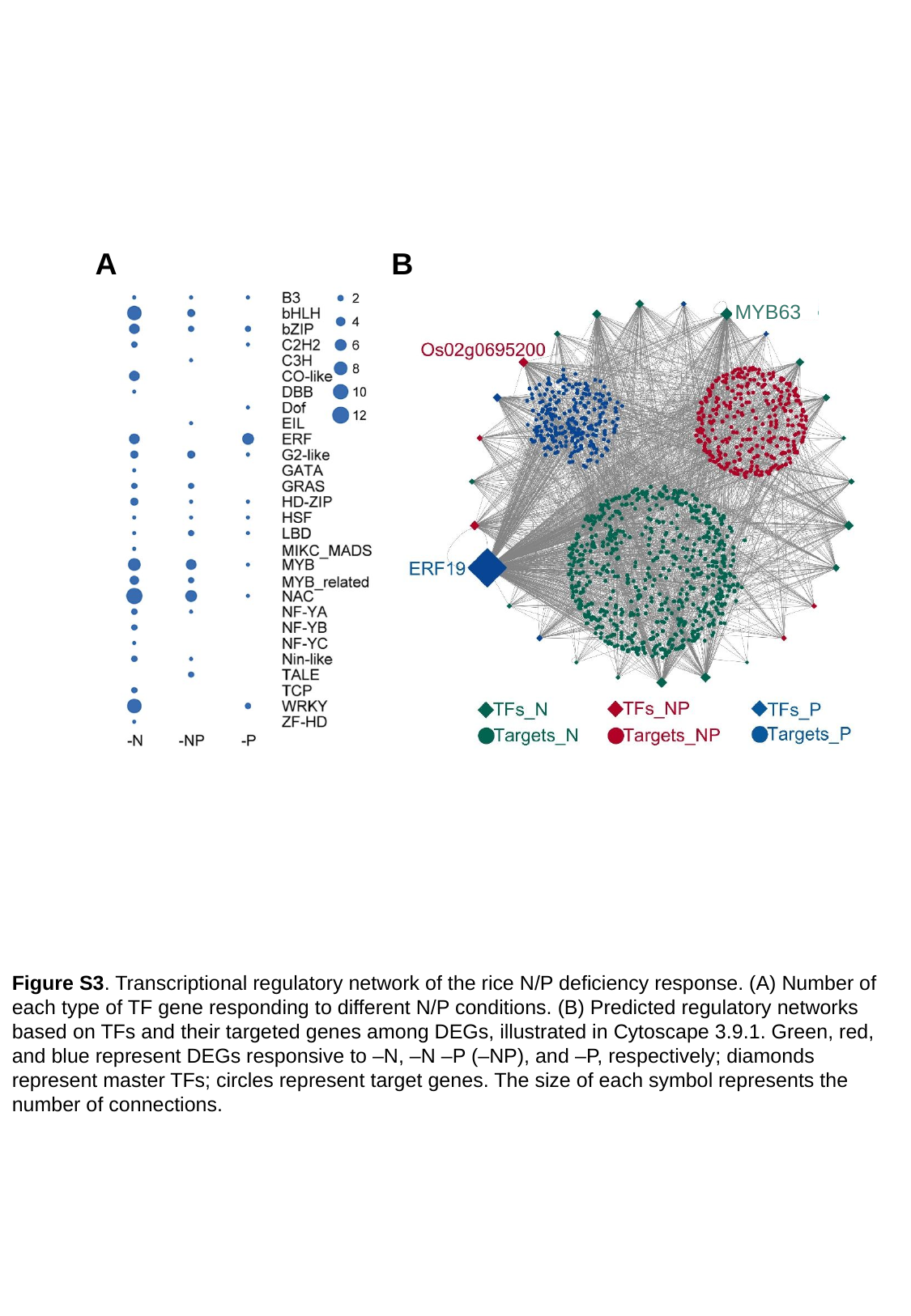

B
A
MYB63
Figure S3. Transcriptional regulatory network of the rice N/P deficiency response. (A) Number of each type of TF gene responding to different N/P conditions. (B) Predicted regulatory networks based on TFs and their targeted genes among DEGs, illustrated in Cytoscape 3.9.1. Green, red, and blue represent DEGs responsive to –N, –N –P (–NP), and –P, respectively; diamonds represent master TFs; circles represent target genes. The size of each symbol represents the number of connections.

## Slide 4
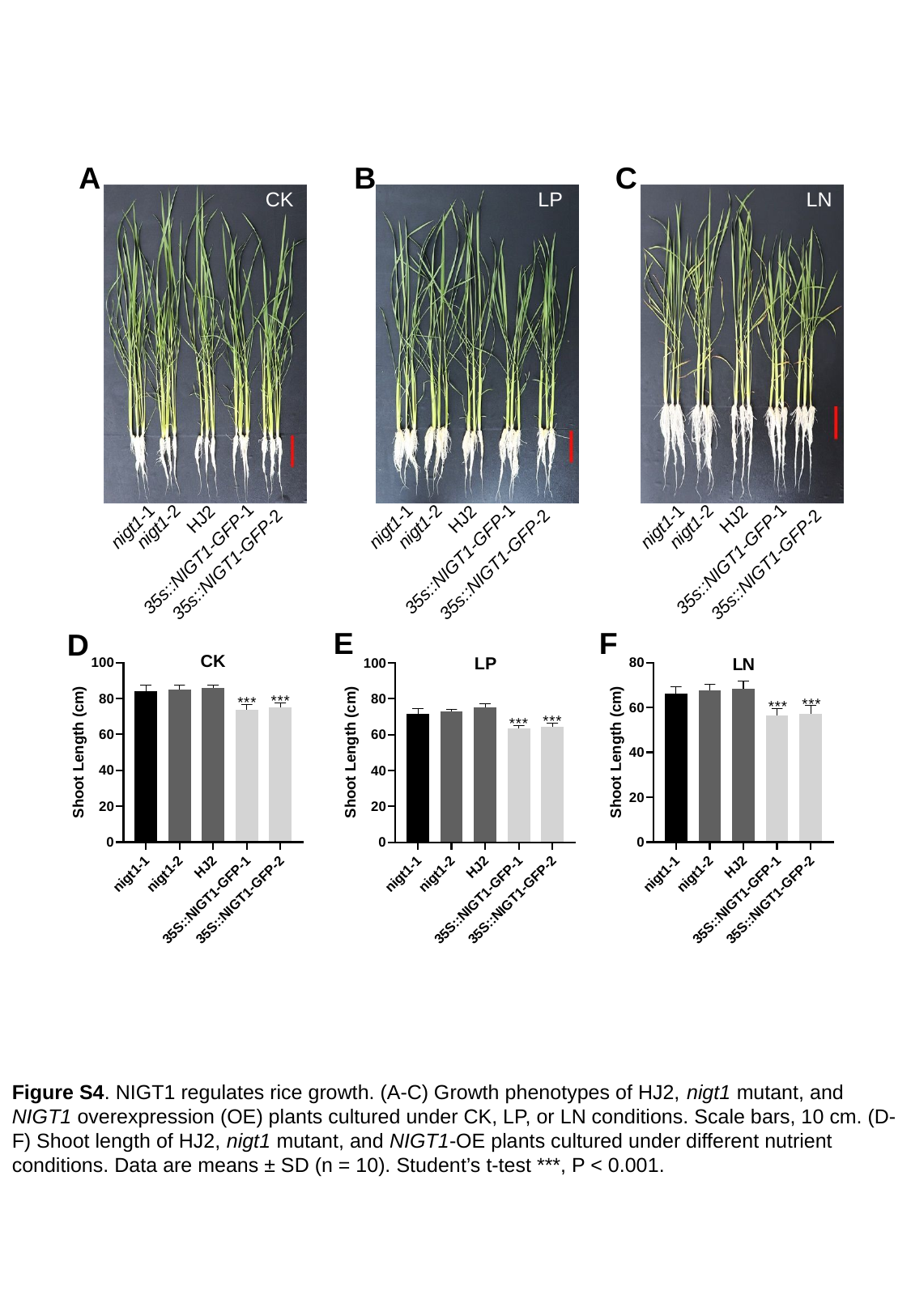

C
A
B
CK
LP
LN
HJ2
HJ2
HJ2
nigt1-1
nigt1-2
nigt1-1
nigt1-2
nigt1-1
nigt1-2
35s::NIGT1-GFP-1
35s::NIGT1-GFP-2
35s::NIGT1-GFP-1
35s::NIGT1-GFP-2
35s::NIGT1-GFP-1
35s::NIGT1-GFP-2
E
F
D
Figure S4. NIGT1 regulates rice growth. (A-C) Growth phenotypes of HJ2, nigt1 mutant, and NIGT1 overexpression (OE) plants cultured under CK, LP, or LN conditions. Scale bars, 10 cm. (D-F) Shoot length of HJ2, nigt1 mutant, and NIGT1-OE plants cultured under different nutrient conditions. Data are means ± SD (n = 10). Student’s t-test ***, P < 0.001.

## Slide 5
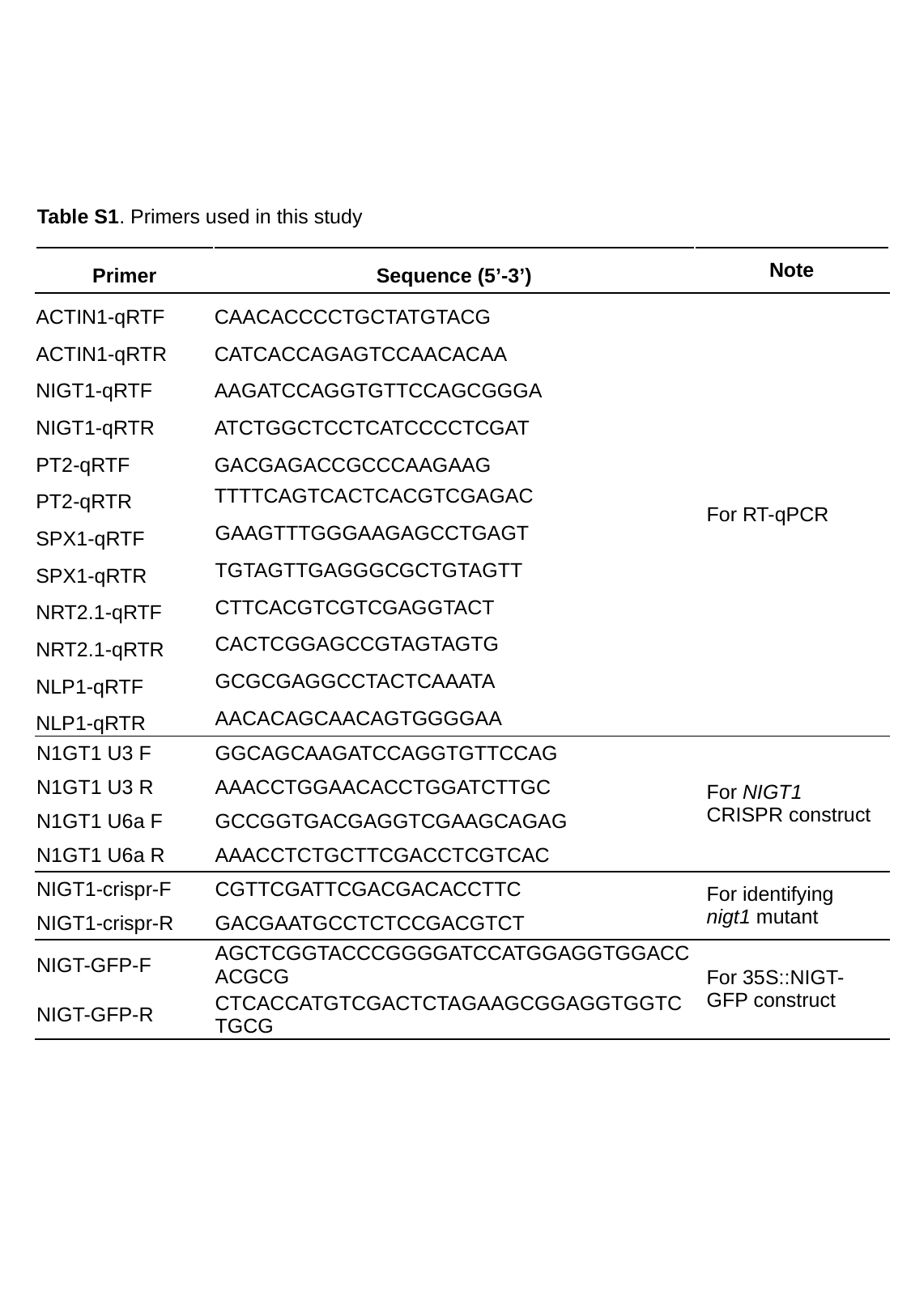

Table S1. Primers used in this study
| Primer | Sequence (5’-3’) | Note |
| --- | --- | --- |
| ACTIN1-qRTF | CAACACCCCTGCTATGTACG | For RT-qPCR |
| ACTIN1-qRTR | CATCACCAGAGTCCAACACAA | |
| NIGT1-qRTF | AAGATCCAGGTGTTCCAGCGGGA | |
| NIGT1-qRTR | ATCTGGCTCCTCATCCCCTCGAT | |
| PT2-qRTF | GACGAGACCGCCCAAGAAG | |
| PT2-qRTR | TTTTCAGTCACTCACGTCGAGAC | |
| SPX1-qRTF | GAAGTTTGGGAAGAGCCTGAGT | |
| SPX1-qRTR | TGTAGTTGAGGGCGCTGTAGTT | |
| NRT2.1-qRTF | CTTCACGTCGTCGAGGTACT | |
| NRT2.1-qRTR | CACTCGGAGCCGTAGTAGTG | |
| NLP1-qRTF | GCGCGAGGCCTACTCAAATA | |
| NLP1-qRTR | AACACAGCAACAGTGGGGAA | |
| N1GT1 U3 F | GGCAGCAAGATCCAGGTGTTCCAG | For NIGT1 CRISPR construct |
| N1GT1 U3 R | AAACCTGGAACACCTGGATCTTGC | |
| N1GT1 U6a F | GCCGGTGACGAGGTCGAAGCAGAG | |
| N1GT1 U6a R | AAACCTCTGCTTCGACCTCGTCAC | |
| NIGT1-crispr-F | CGTTCGATTCGACGACACCTTC | For identifying nigt1 mutant |
| NIGT1-crispr-R | GACGAATGCCTCTCCGACGTCT | |
| NIGT-GFP-F | AGCTCGGTACCCGGGGATCCATGGAGGTGGACCACGCG | For 35S::NIGT-GFP construct |
| NIGT-GFP-R | CTCACCATGTCGACTCTAGAAGCGGAGGTGGTCTGCG | |

## Slide 6
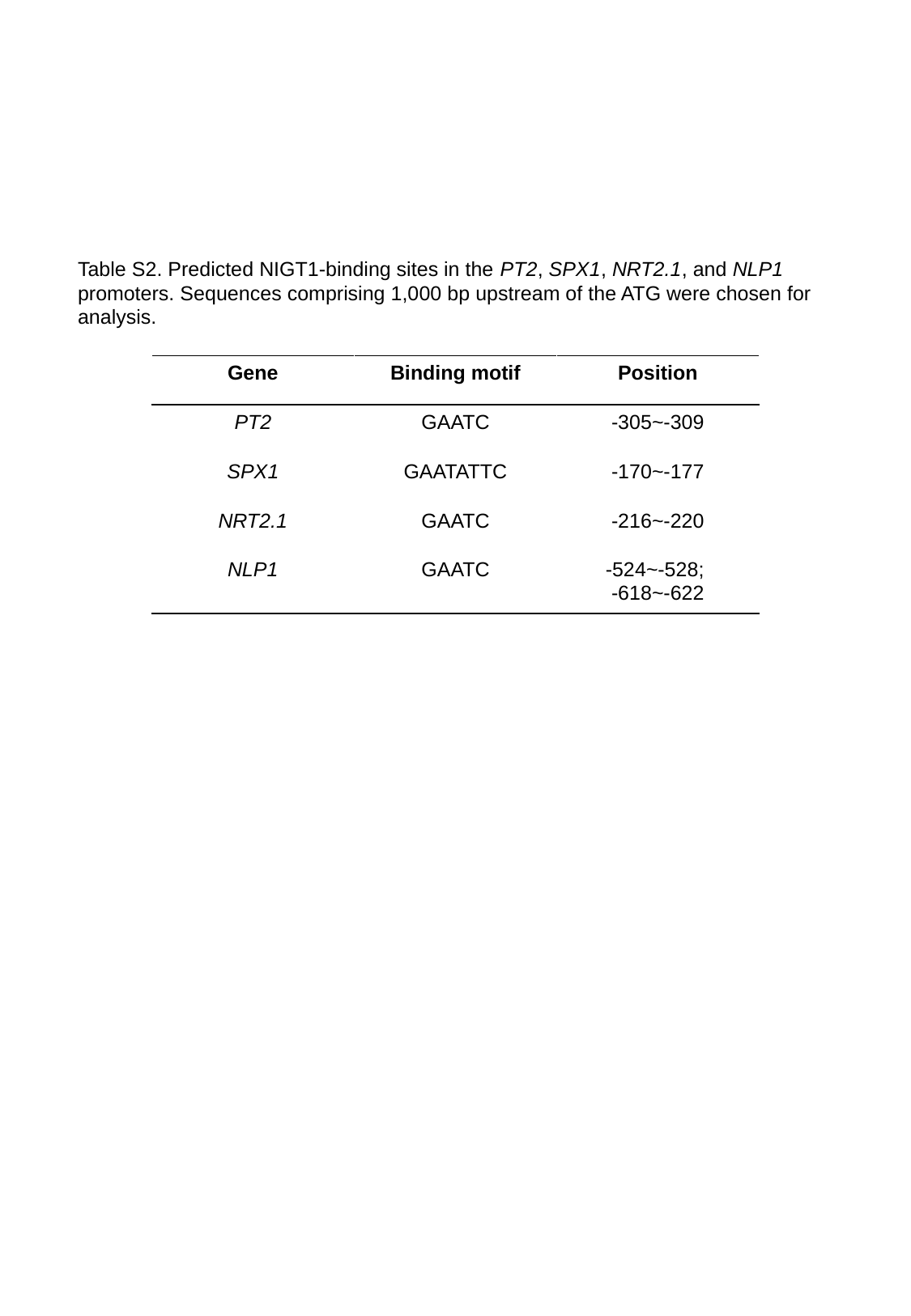

Table S2. Predicted NIGT1-binding sites in the PT2, SPX1, NRT2.1, and NLP1 promoters. Sequences comprising 1,000 bp upstream of the ATG were chosen for analysis.
| Gene | Binding motif | Position |
| --- | --- | --- |
| PT2 | GAATC | -305~-309 |
| SPX1 | GAATATTC | -170~-177 |
| NRT2.1 | GAATC | -216~-220 |
| NLP1 | GAATC | -524~-528; -618~-622 |
